# Research on Digital Twin Modeling and Fault Diagnosis Methods for Rolling Bearings

**DOI:** 10.3390/s25072023

**Published:** 2025-03-24

**Authors:** Jiayi Fan, Lijuan Zhao, Minghao Li

**Affiliations:** 1 School of Mechatronics Engineering, Shenyang Aerospace University, Shenyang 110136, China; 2 College of Mechanical Engineering, Liaoning Technical University, Fuxin 123000, China; 3 Liaoning Provincial Key Laboratory of Large-Scale Mining Equipment, Fuxin 123000, China; 4 School of Mechanical Engineering, Shenyang Ligong University, Shenyang 110159, China

**Keywords:** digital twin, rolling bearing, dynamic model, hybrid noise

## Abstract

This paper proposed a digital twin modeling method based on digital twin technology to improve the operational stability of rolling bearings and the accuracy of fault diagnosis methods. A comprehensive digital twin model for the entire lifecycle of rolling bearings was constructed using Modelica language. This model included a multi-state rolling bearing digital twin and integrated twin models for both the bearing drive and load ends. The model employed hybrid noise component to simulate the bearing’s actual operating state and degradation process with high fidelity. Based on experimental lifecycle data from the laboratory, the rolling bearing full-life digital twin integrated model parameters were updated. Through the degradation components of the digital twin, the twin data of the rolling bearing was generated. By combining the twin data with actual measurement data, this approach addresses the limitations of traditional methods in the absence of data for bearings, providing reliable technical support for intelligent maintenance and fault diagnosis methods for rolling bearings.

## 1. Introduction

Rolling bearings are one of the key components widely used in mechanical equipment. They are characterized by low friction and high operational efficiency, often called industrial joints. A rolling bearing consists primarily of an outer ring, inner ring, cage, and rolling elements. Bearing failures account for nearly 45–55% of all equipment failures [1], and when a failure occurs, it can lead to unplanned downtime in the entire mechanical system, resulting in significant economic losses. Accurate fault diagnosis of rolling bearings can predict the health status of the bearings in advance, allowing for the development of a reasonable maintenance plan based on the health status. This helps to ensure smooth equipment operation and reduces losses due to unscheduled downtime. Therefore, accurate fault diagnosis of rolling bearings is of great significance and value in guaranteeing safe equipment operation and improving factory production efficiency.

To achieve accurate fault diagnosis of rolling bearings, scholars domestically and internationally have researched two main aspects: rolling bearing fault diagnosis and digital twin model establishment. In the field of rolling bearing fault diagnosis, in 2012, Kim H used SVM to estimate health state probability during the bearing degradation process. Effective features were selected by employing a distance-based evaluation criterion between all attributes, which improved the classification accuracy and diagnostic precision of SVM in bearing fault diagnosis [2]. In 2013, MURUGANATHAM B used singular-value decomposition and feedforward backpropagation artificial neural networks (ANNs) to estimate different bearing fault categories for bearings of various sizes [3]. In 2015, TIAN Y employed a combination of neural networks and empirical mode decomposition (EMD) to diagnose bearing faults based on vibration signals. The author selected key intrinsic mode functions and input the chosen features into an ANN for fault identification [4]. Similarly, ALI J proposed a framework using a feedforward backpropagation ANN with two hidden layers to classify faults in rolling elements, inner and outer rings at normal, degraded, and severe fault stages [5]. The application of deep neural networks has advanced rolling bearing fault diagnosis to some extent. Convolutional neural networks (CNN) are divided into 1D-CNN and 2D-CNN, enabling them to process both linear data and image data. In 2019, LI X proposed fault diagnosis technology based on deep generative adversarial networks (GANs) and cross-domain transfer learning, which provided effective cross-domain diagnostic results when test data were not applicable to the training set [6]. In 2020, ZHANG J converted raw bearing fault signals into 2D images and used a CNN to extract features from the converted 2D images [7]. In 2022, a category-imbalanced privacy-preserving federated learning framework was proposed for fault diagnosis in distributed wind turbines, aiming to improve the diagnostic accuracy of distributed machines without the need for data transmission [8]. The authors of [9] proposed instantaneous spectral entropy and continuous wavelet transform for anomaly detection and fault diagnosis, divorced from transmission vibration time history. In 2023, GUO Y demonstrated that a CNN could directly integrate with visual methods to diagnose bearing faults by learning image information [10]. TAO T combined multi-wavelet packet decomposition with a CNN, enabling the model to extract and learn useful time–frequency-domain information of the signal at multiple scales. This approach improved accuracy by 13.19% compared to conventional CNN methods [11]. To enhance data collection and transmission capabilities, a novel event-triggered federated learning framework was proposed for decentralized fault diagnosis of offshore wind turbines [12]. In 2024, WU J obtained a 2D time–frequency map from raw bearing vibration signals using continuous wavelet transform and utilized DDPMs (Denoising Diffusion Probabilistic Models) to augment small sample sizes. The experimental results showed that this method effectively increased fault diagnosis accuracy, demonstrating its effectiveness and superiority [13].

To address issues such as the difficulty in determining thresholds, roughness, and operability in the cross-correlation integral for rolling bearings, a Dynamic Difference Index was proposed [14].

In 2025, ZOU* conducted experimental verification of the proposed method using a cross-condition rolling bearing fault dataset and compared it with single-domain and other multi-domain diagnostic methods, proving the effectiveness and superiority of the proposed approach [15]. Babu TN combined wavelet analysis with machine learning (ML) algorithms to improve the accuracy and reliability of roller bearing defect diagnosis [16].

Research on digital-twin-technology-based bearings has become a hotspot in fault diagnosis. Many scholars have conducted studies on rolling bearings using digital twin technology. In 2022, Tuo YT utilized digital twin technology to obtain real-time perception information of rolling bearings and developed a digital twin model that reflects operational condition changes in real time [17]. In 2023, MA LM established a digital twin model for a bearing test bench, achieving fault diagnosis of bearings through simulation and physical objects [18]. ZHANG C proposed a method for constructing health indicators of rotating machinery driven by digital twins to address the challenge of accurately predicting the remaining useful life (RUL) of rotating machinery under small-sample-data conditions [19]. In 2024, HUANG XF proposed an incremental learning method based on multi-fidelity information fusion for continuous fault diagnosis of critical rolling bearing failures. The experimental results indicated that this method could effectively diagnose different health conditions over time [20]. SHI HT introduced a model–data combination-driven digital twin (MDCDT) model for fault diagnosis in rolling bearings with limited sample data. The experiments showed that MDCDT could generate virtual data for fault diagnosis, significantly improving diagnostic accuracy [21]. In 2025, ZHANG YC proposed a digital-twin-driven framework with an average diagnostic accuracy of 84.39%, over 10% higher than the current advanced domain adaptation methods [22]. MING Z enhanced data using generated digital twin data and proposed a frequency filter subdomain adaptation network to achieve feature transfer between twin data and measured data [23].

A digital twin is a real-time virtual representation of a physical entity created through digital technologies, capable of dynamically reflecting its state and enabling prediction and optimization. Digital twins are widely used in mechanical engineering for equipment health monitoring, fault diagnosis, predictive maintenance, and performance optimization. Digital twin technology has been extensively applied in applications such as rolling bearings, wind turbine systems, and industrial robots, significantly improving operational efficiency and reliability. The aforementioned rolling bearing fault diagnosis methods based on digital twin technology still require sample data from actual operating conditions to extract evolutionary feature patterns and interact with simulation data. In cases of small and limited sample sizes, the accuracy of fault diagnosis by the models needs further improvement. This paper constructs a digital twin integrated model for rolling bearings based on the five-dimensional digital twin system framework. The model includes a multi-state digital twin model for rolling bearings, integrates twin models for the bearing’s drive end and load end, and incorporates a hybrid noise component, enabling high-fidelity simulation of the actual operating conditions of the bearings. A digital twin integrated model for rolling bearings was constructed based on the Modelica language. This model encompasses a multi-state digital twin model for rolling bearings and integrates twin models for both the driving and load end of the bearings. Additionally, it incorporates hybrid noise component, enabling high-fidelity simulation of the actual operational states and degradation processes of the bearings. Through this model, ample and reliable twin data for bearings were generated. Based on the existing full-measured bearing data, the full-life digital twin integrated model parameters for rolling bearings were updated. The parameter degradation component of the twin model was used to generate twin data for the rolling bearings. By combining the twin data with actual measurement data, the shortcomings of traditional methods in bearing fault diagnosis under data scarcity were addressed, offering reliable technical support for the intelligent maintenance and optimization of rolling bearings.

## 2. Rolling Bearing Digital Twin Modeling

### 2.1. Digital Twin System Framework for Rolling Bearings

Based on a five-dimensional digital twin system framework and a rotating machinery vibration and fault test bench as the research platform, a rolling bearing digital twin integrated model is constructed with digital twin technology. Modelica is an open physical modeling language used to describe the dynamic behavior of physical systems. Its modular design and open standards make models easy to reuse and extend. In digital twin research, Modelica demonstrates excellent applicability in high-precision modeling, multi-physics coupling capabilities, and real-time data updating, enabling a dynamic reflection of the physical entity’s state. Using a deep groove ball bearing with nine rolling elements as an example, the Modelica modeling method is employed to establish digital twin models for the deep groove ball bearing under different health states (healthy, inner-outer ring defects, rolling element defects). The digital twin model related to the motor-shaft system, hydraulic system, and random noise are also created [24,25,26]. The connections between the digital twin models are established, adopting a hybrid modeling approach to create a digital twin integrated model library for rolling bearings [27,28].

**Figure 1:**
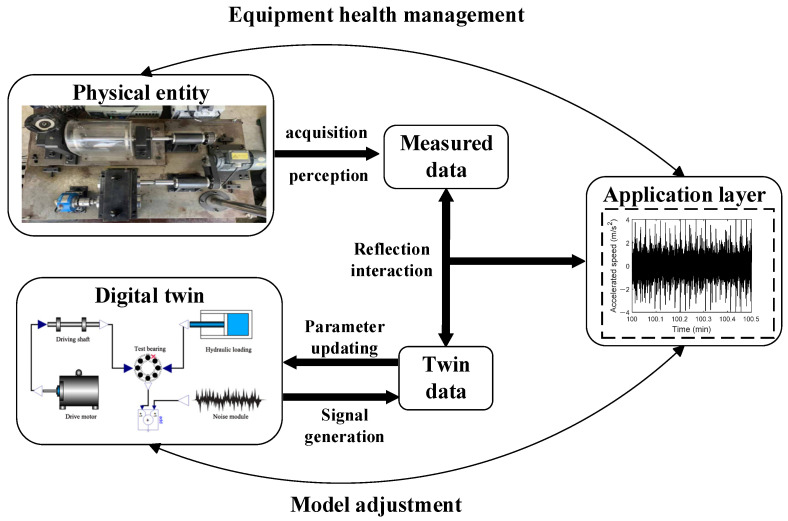
Construction scheme of digital twin system for rolling bearings.

The QPZZ-II rotating machinery vibration and fault test bench is used as the physical entity for research. It is an experimental device specifically designed to simulate and analyze the vibration characteristics and fault diagnosis of rotating machinery under various conditions. The Modelica method is employed to construct the twin model based on the dynamic relationships of the physical entity. This language adopts an equation-based approach to describe real physical systems, enabling comprehensive performance analysis and evaluation of modern complex engineering systems, and is applicable to most engineering fields. The data connection layer of the rolling bearing digital twin system serves as the core hub of the entire system. It is responsible for tightly linking various dimensions of the system—physical entity, digital twin, application layer, and twin information—through various communication protocols, achieving efficient data acquisition and transmission. In the architecture of the digital twin system, the application layer, with its intuitive representation, forms the interface for direct user interaction. This layer is the primary channel for users to obtain information and execute operations. Through this dimension, users interact with the system, receive feedback, and implement control. The structure of the final digital twin integrated model for rolling bearings is shown in Figure 1.

In this system, the motor–shaft system provides the rotational speed information, the hydraulic drive system provides the load information, and the hybrid noise module is added during the actual measurement process to simulate the information influenced by measurement errors, uncontrollable environmental factors, and other effects, as recorded by the accelerometer. Through this structure, a complete digital twin integrated model for rolling bearings is built, forming a mapping relationship with the actual operation process of the physical components and the information acquisition process.

### 2.2. Rolling Bearing Fault and Degradation Analysis and Signal Selection Method

The fault vibration signals of rolling bearings and their mating components are typically concentrated in the high-frequency resonance region. Vibration signals of rolling bearings are usually collected using accelerometers, which eliminate interference and reveal vibration characteristic information caused by faults. An effective health state model can be established by analyzing the features and trends in the vibration signals. The relevant parameters monitored are shown in Table 1.

## 3. Digital Twin Integrated Model Construction for Rolling Bearings

### 3.1. Bearing Dynamics Analysis

A healthy bearing achieves dynamic balance during stable operation. However, once defects occur on the contact surfaces, a series of impacts are generated. Taking deep groove ball bearings as an example, a dynamic analysis of the rolling bearing is performed, focusing on the nonlinear contact forces between rigid bodies and constructing its dynamic model. Finally, the dynamic characteristics of the bearing under inner and outer ring defects and rolling element defects are analyzed.

**Figure 2:**
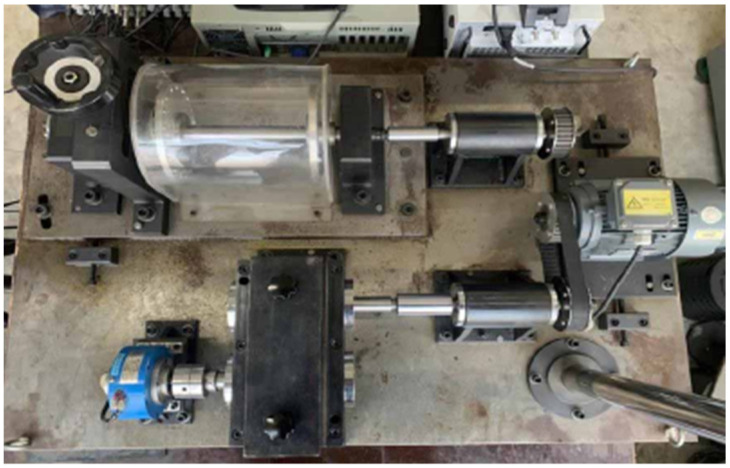
Rotating machinery vibration and fault test bench.

This study uses the rotating machinery vibration and fault test bench at the Key Laboratory of Advanced Manufacturing Technology and Equipment of Liaoning Province as the physical entity for research, as shown in Figure 2.

The test bench primarily comprises the following components: a 0.55 kW AC variable-frequency drive motor, bearing, gearbox, shaft, unbalanced rotor, and speed controller. This test bench is specifically designed to simulate and analyze rotating machinery’s vibration characteristics and fault diagnosis under various conditions. It has multiple functions, enabling rapid simulation of different types of mechanical faults, ansd speed variation simulations, as well as data collection and analysis by adjusting counterweights, changing the mounting positions of adjustable parts, and combining components flexibly.

**Figure 3:**
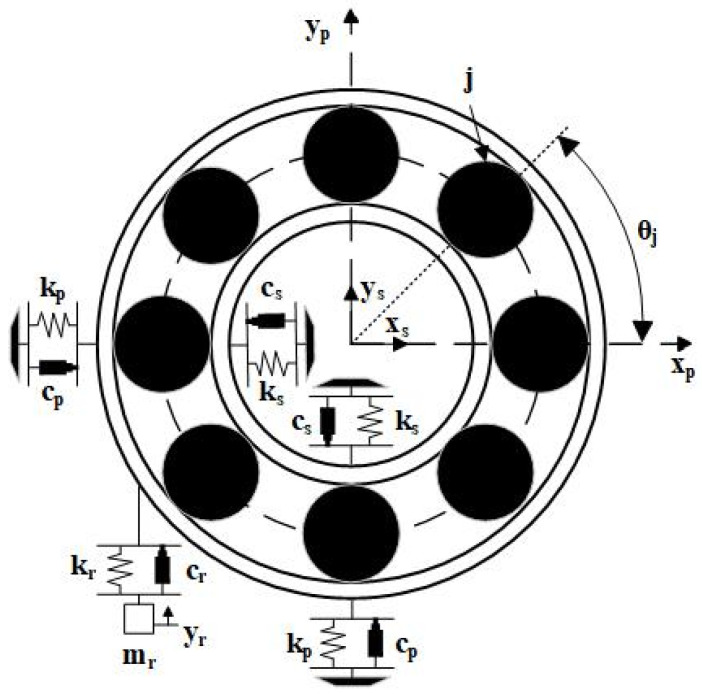
Five-degree-of-freedom rolling bearing model.

The five-degree-of-freedom (5-DoF) bearing dynamics model describes the nonlinear dynamic behavior of the bearing, as shown in Figure 3.

Twin models for healthy bearings, inner ring defects, outer ring defects, and rolling element defects are constructed [29].

In the 5-DoF model, the 4-DoF represents the horizontal and vertical directions of the inner and outer rings, while the 1-DoF in the vertical direction represents the bearing’s high-frequency response. This model is formulated as a spring–damper–mass system, as proposed by Cui [30]. The model adheres to the following five assumptions:

(1) All components are modeled using the concentrated mass method;
(2) There is no inertia moment;
(3) There are no geometric errors between the rolling elements and the contact surfaces of the inner and outer rings;
(4) The contact between the rolling elements and the inner and outer rings follows Hertzian theory;
(5) All damping is assumed to be linear viscous.

The dynamic differential equation of the model is as follows:

(1) $$\begin{array}{l} m_{s}{\ddot{x}}_{s}+R_{s}{\dot{x}}_{s}+K_{s}x_{s}+f_{x}=0\\ m_{s}{\ddot{y}}_{s}+R_{s}{\dot{y}}_{s}+K_{s}y_{s}+f_{y}=F-m_{s}g\\ m_{p}{\ddot{x}}_{p}+R_{p}{\dot{x}}_{p}+K_{p}x_{p}-f_{x}=0\\ m_{p}{\ddot{y}}_{p}+(R_{p}+R_{r}){\dot{y}}_{p}+(K_{p}+K_{r})y_{p}-R_{r}{\dot{y}}_{r}-K_{r}y_{r}-f_{y}=-m_{p}g\\ m_{r}{\ddot{y}}_{r}+R_{r}({\dot{y}}_{r}-{\dot{y}}_{p})+K_{r}(y_{r}-y_{p})=-m_{r}g \end{array}$$

$m_{s},m_{p},m_{r}$ represent the masses of the inner race, outer race, and the resonator, respectively; $R_{s},R_{p},R_{r}$ represent the damping values of the inner race, outer race, and the resonator, respectively; $K_{s},K_{p},K_{r}$ represent the stiffness values of the inner race, outer race, and the resonator, respectively; x,y represent the displacement in each degree of freedom; $f_{x}$ and $f_{y}$ are the contact forces along the x-axis and y-axis, with F representing the external load.

When a local fault occurs in the inner or outer race of the rolling element bearing, the rolling element moving over the local fault point will release additional deformation* $\Delta \delta_{j}$. The deformation of the *j*-th rolling element as it enters the spalling zone is expressed as follows:

(2) $$\delta_{j}=(x_{s}-x_{p})\cos\varphi_{j}+(y_{s}-y_{p})\sin\varphi_{j}-\Delta \delta_{j}$$

In the formula, $\Delta \phi_{d}$ represents the angular range of the defect, and B* represents the defect’s width. The relationship between the two can be expressed as follows:

(3) $$\sin\left(\frac{1}{2}\Delta \phi_{d}\right)=\frac{B}{D_{b}+D_{p}}$$

Assuming the defect depth is $C_{d}$, the deformation $\Delta \delta_{j}$ due to the defect only depends on the angular position. The deformation will occur and exist only when the angular position of the rolling element is between $\phi_{d}$ and $\phi_{d}+\Delta \phi_{d}$. The relationship can be expressed as follows:

(4) $$\Delta \delta_{j}=\left\{\begin{array}{ll} C_{d}, & \varphi_{d}\leq \theta_{j}\leq \varphi_{d}+\Delta \varphi_{d}\\ 0, & \mathrm{else} \end{array}\right.$$

The defect position on the outer or inner race changes according to different rules. For the outer race, the defect is fixed at the initial defect angle $\phi_{do}$. However, when the inner race of the rolling bearing rotates, the defect position on the inner race is not fixed at the initial position $\phi_{di}$, but changes over time. Therefore, this process is modeled as follows:

(5) $$\varphi_{d}=\left\{\begin{array}{l} \varphi_{do},\;\mathrm{Outer}\;\mathrm{ring}\;\mathrm{fault}\\ \omega_{s}t+\varphi_{di},\;\mathrm{Inner}\;\mathrm{ring}\;\mathrm{fault} \end{array}\right.$$

Unlike the faults on the inner and outer races of the rolling bearing, when a defect occurs on a rolling element, the defect spins with the rolling element speed $\omega_{b}$. The position of the defect is expressed as follows:

(6) $$\phi_{s}=\omega_{b}t+\varphi_{s_{ini}}$$

The rolling element speed can be calculated from the shaft speed $\omega_{b}$ as follows:

(7) $$\omega_{b}=\frac{\omega_{s}}{2}\frac{D_{p}}{D_{b}}\left[1-{\left(\frac{D_{p}}{D_{b}}\cos\alpha \right)}^{2}\right]$$

The defect on the rolling element periodically contacts the inner and outer rings. Due to the different inner and outer ring curvature radii, the same defect span angle will result in different angular widths $\Delta \phi_{d}$. The angular widths of the inner and outer ring defects can be calculated as follows:

(8) $$\Delta \phi_{bo}=\Delta \phi_{d}\frac{D_{b}}{D_{o}}$$

(9) $$\Delta \phi_{bi}=\Delta \phi_{d}\frac{D_{b}}{D_{i}}$$

The acronyms, $D_{o}$ and $D_{i}$ represent the diameters of the outer and inner rings, respectively.

When the deformation caused by the fault defect appears only on the rolling element with the defect (the *j*-th rolling element), the contact deformation of the defective rolling element is given by:

(10) $$\Delta \delta_{j}=\left\{\begin{array}{l} 0,\;j\neq k\\ C_{d},\;j=k \end{array}\right.$$

### 3.2. Hybrid Noise Module Component Analysis

A hybrid noise component is a collection of multiple noise components simultaneously present in bearing vibration signals or other monitoring data. It is modeled by combining Gaussian and band-limited white noise. Hybrid noise plays a significant role in noise modeling for signal processing and control systems, effectively describing the randomness and uncertainty in the digital twin systems of rolling bearings. The hybrid noise component of the rolling bearing full-life digital twin integrated model is used to fit errors generated during the actual detection process and account for environmental noise’s influence.

Gaussian noise is a type of random noise with a normal distribution widely used in wireless communication and signal processing. Its probability density function can be expressed as follows:

(11) $$p(x)=\frac{1}{\sqrt{2\pi \sigma^{2}}}e^{-\frac{{\left(x-\mu \right)}^{2}}{2\sigma^{2}}}$$

In the formula, μ represents the mean; $\sigma^{2}$ represents the variance. Band-limited white noise refers to random noise with a uniform power spectral density within a specific frequency range. It is commonly used as a model to describe random disturbances in a system. Its power spectral density can be expressed as follows:

(12) $$S(f)=\left\{\begin{array}{c} N_{0}\;|f|\leq B\\ 0\;|f|>B \end{array}\right.$$

In the formula, $N_{0}$ represents the power spectral density of the noise; B represents the bandwidth limit. The correlation between Gaussian and band-limited white noise can be described using the autocorrelation function. The autocorrelation function of Gaussian noise is given by:

(13) $$R(\tau )=\sigma^{2}\delta (\tau )$$

In the formula, δ(τ) is the Dirac delta function, representing the complete independence of Gaussian noise samples over time.

For band-limited white noise, the autocorrelation function can be expressed as follows:

(14) $$R(\tau )=\frac{N_{0}}{2}rect(\frac{\tau }{B})$$

where rect(⋅) is the rectangular function, indicating the correlation within the bandwidth B.

## 4. Operation of the Digital Twin and Model Update

### 4.1. The Design Flowchart of Model Update

**Figure 4:**
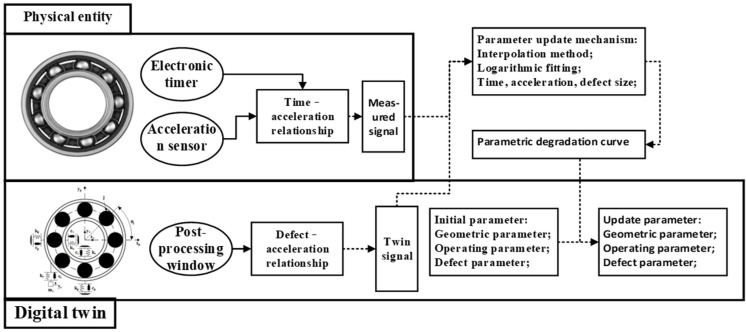
Updating mechanism of bearing digital twin degradation parameters.

To ensure that the dynamic twin model established within the digital twin framework accurately reflects the actual vibration response, a degradation parameter update mechanism is incorporated into the digital twin model, as shown in Figure 4. This method uses actual measurement data from the rolling bearing to adjust the parameters of the dynamic twin model.

### 4.2. Construction of Digital Twin System

**Figure 5:**
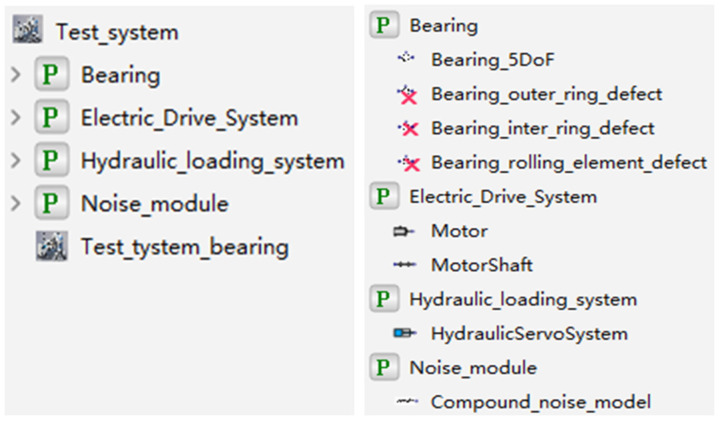
Digital twin system library structure of rolling bearing.

A comprehensive digital twin integrated model for rolling bearings is constructed based on digital twin technology, using deep groove ball bearings as an example. A digital twin model for deep groove ball bearings under different health states is established using the Modelica modeling approach. Additionally, a digital twin model for the motor–shaft system, hydraulic system, and random noise is developed. Interfaces for the inputs and outputs of each component are created, connecting them into a complete digital twin model system. By running the twin model with given parameters and updating defect parameters using fitted degradation curves, fault data and full-life degradation data are generated. A hybrid modeling approach combining block diagram modeling and acausal modeling is employed to build a digital twin integrated model library for the full lifecycle of rolling bearings, as illustrated in Figure 5.

**Figure 6:**
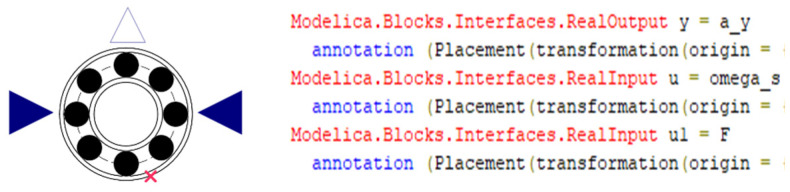
Bearing twin model with added interface.

After the sub-component twins are constructed, the twin model of the bearing under test remains an independent model. Interfaces are added to each sub-component model to connect the twin models into a complete system. Taking the rolling bearing twin model as an example, as shown in Figure 6, two input interfaces are added, along with a triangular output interface to transmit vibration signal information.

Using the results from the healthy state, outer race fault state, and the updated outer race degradation state as examples, the operation of the digital twin outputs signals for the healthy and outer race fault states.

### 4.3. Healthy State

**Figure 7:**
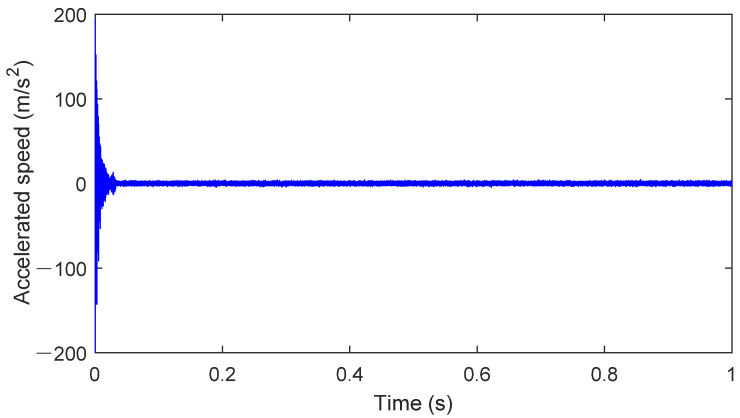
Bearing health operation results.

The packaged healthy bearing model is dragged into the system, and the interfaces are connected. After checking the parameters of all system components, the simulation time interval is set to 0–1 s in the simulation setup interface. The integration algorithm used is Dassl, with an integration accuracy of 0.0001. The speed is set to 1750 rpm, the external load is set to 900 N, and the sampling frequency is set to 12 kHz. The results of the simulation are shown in Figure 7.

**Figure 8:**
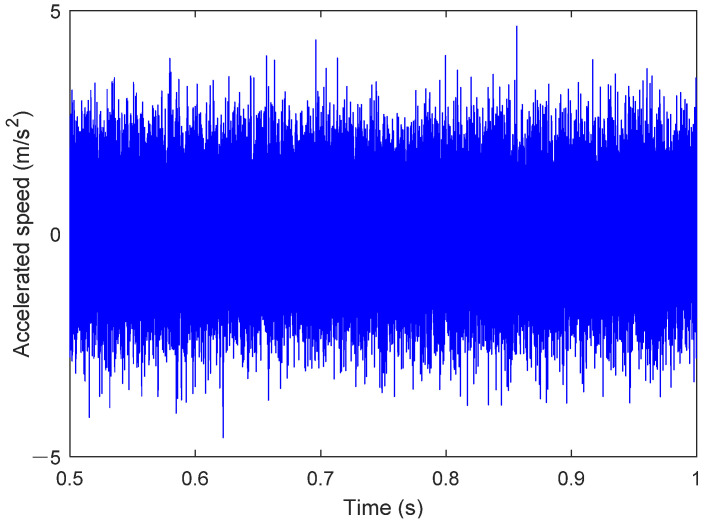
Bearing health operation results (0.5–1 s).

The signal in Figure 7 represents the y-axis acceleration vibration signal obtained from the operation of the digital twin. For the physical rolling bearing test system, a magnetically attached accelerometer placed at the corresponding bearing seat position can be used to measure the vibration signal of the healthy bearing. The results for stable operating conditions are shown in Figure 8. As seen in Figure 8, when the bearing is healthy, its operating state typically manifests as stable noise signals. The characteristics of the noise signal indicate that friction and lubrication conditions are good, and the spectral analysis will show conventional frequency components, without abnormal peaks or an increase in frequency components. Additionally, the stability of the vibration signal also suggests the absence of obvious faults or wear.

### 4.4. Outer Race Fault State

**Figure 9:**
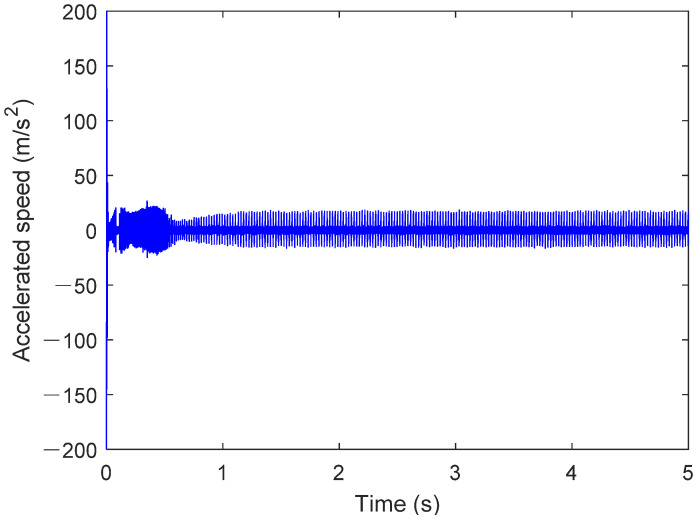
Operation result of bearing outer ring failure.

In the simulation settings interface, the simulation time interval is set to 0–5 s and the Dassl integration algorithm is used with an integration accuracy of 0.0001. The rotational speed is set to 1750 rpm, the external load is 900 N, and the sampling frequency is 12 kHz. The signal in Figure 9 represents the y-axis acceleration vibration signal obtained from the operation of the digital twin. The results are shown in the figure below.

**Figure 10:**
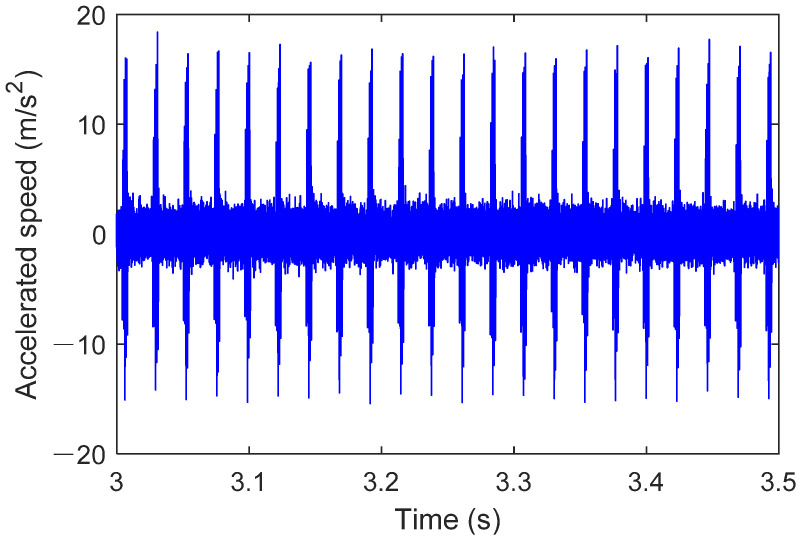
Operation result of bearing outer ring failure (3–3.5 s).

The results in Figure 9 show that the operation stabilizes after 1.2 s, which is related to the motor drive and hydraulic loading process. In the steady state, the outer race fault bearing causes specific fault frequency components to appear in the acceleration signal. The amplitude variation is usually proportional to the severity of the fault, reflecting the increased impact and vibration. At the same time, the acceleration signal often exhibits a periodic waveform, which is closely related to the rotation of the outer race and the impact of the rolling elements. Distinct impact pulses may appear in the signal, and the repetition interval of these pulses is consistent with the rolling element’s motion cycle. Outer race faults can lead to nonlinear components in the acceleration signal, indicating dynamic imbalance and friction instability caused by the fault. Figure 10 shows the operating results of the outer race fault bearing from 3 s to 3.5 s.

### 4.5. Parameter Update and Degradation Signal Generation

For example, the rolling bearing digital twin model shows its geometric parameters and some defect parameters in Table 2.

**Figure 11:**
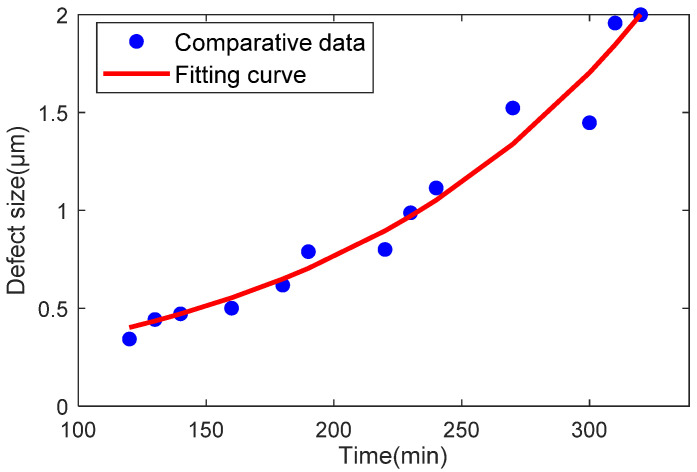
Regression curve of fitted parameters.

The parameter update method considers the multi-feature similarity of the measured signals. By accounting for the similarity between the “time-acceleration information” of the physical model and the “defect size-acceleration information” of the twin model, the method integrates both sets of information to obtain the “time-defect size” relationship for bearing degradation. This relationship is then mapped to the defect parameters of the digital twin model. A curve fitting method is used to generate the parameter degradation curve, as shown in Figure 11.

The defect parameters of the dynamic twin model are updated to ensure a good match between the twin data and the actual data. This ensures that the twin model can generate reliable outputs based on the parameter update method. The exponential function model proposed by Diwang R [31] is used to fit the parameters, as shown in the following equation:

(15) $$A(t)=\zeta A_{0}e^{-B_{\zeta }t}$$

where $A_{0}$ is the initial amplitude, m/s^2^;$B_{\zeta }$ is the decay parameter; t is time, s; ζ is the decay rate (0 < ζ < 1).

**Figure 12:**
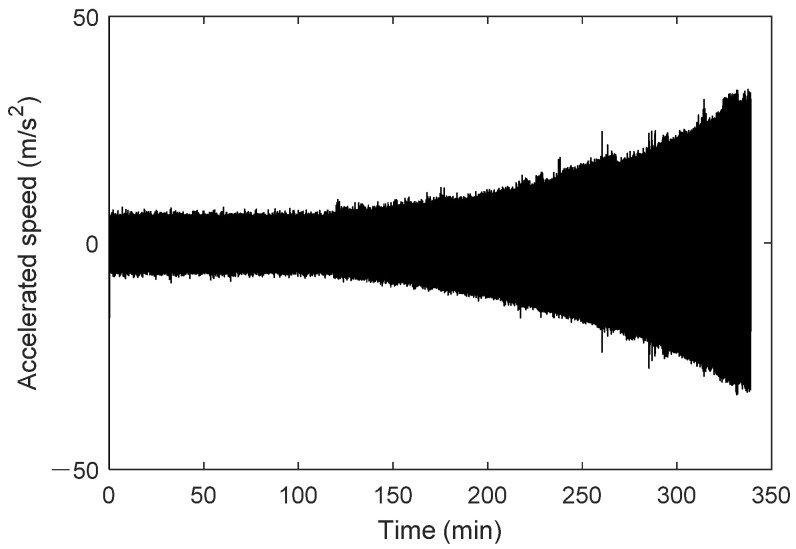
Bearing life twin signal with parameter degradation mechanism.

Taking the outer ring degradation state as an example for parameter update, the twin-generated signal is used. The outer ring degradation model is adjusted by setting the parameter (additional defect deformation) as the fitted degradation curve obtained through the parameter update method. The updated module is then dragged into the system, interfaces are connected, and the system component parameters are checked. After setting them in the simulation interface, the resulting twin signal is shown in Figure 12.

As shown in Figure 12, the generated twin signal of the rolling bearing exhibits a clear degradation trend. In the first half, the signal remains stable with a small amplitude and is dominated by random noise, indicating that the rolling bearing is healthy. Later, the signal amplitude gradually increases, accompanied by significant fluctuations, signaling that the rolling bearing has entered the degradation phase. Throughout the degradation process, the vibration amplitude of the signal increases over time, which aligns with the physical characteristics of bearing degradation and reflects the characteristic increase in the signal’s acceleration value fluctuation as the degradation progresses.

## 5. Digital Twin Signal Validation Experiment

The digital twin signal validation experiment focuses on verifying the usability of the digital twin system. It primarily involves a comparative analysis of the fault and degradation signals generated by the twin system from a signal analysis perspective.

### 5.1. Experimental Data and Digital Twin Model Parameters

Before starting the experiment, data preparation and preprocessing are required. A suitable measured signal dataset should be selected, and the collected vibration signals should be denoised to extract relevant fault feature information. The parameters of the digital twin model need to be adjusted according to different physical entities, including modifying the dimensions of the bearing’s inner and outer rings, stiffness, rolling element dimensions, and quantity. This ensures that the digital twin model’s running results are more similar to the measured signals, highlighting fault feature information and degradation information. By running the digital twin model, vibration signals of rolling bearings under different fault states and full-life signals during the degradation process can be obtained. After preprocessing and feature extraction, these signals can form the training set for the experiment. The digital twin signal validation experiment primarily verifies the effectiveness of the digital twin signals. Representative fault signals are generated by simulating outer ring and inner ring damage, and their performance in fault monitoring is validated through frequency-domain analysis and time-domain analysis.

**Figure 13:**
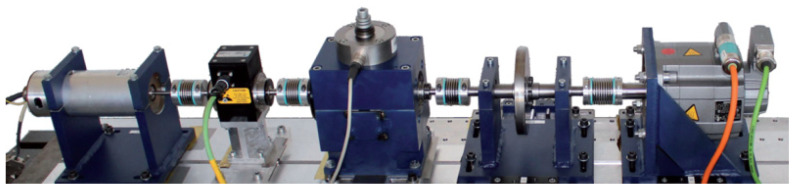
Bearing experimental equipment of Paderborn University, Germany.

The experiment uses the bearing dataset from the University of Paderborn, Germany. The experimental setup is shown in Figure 13. The test rig consists of several modules (from left to right): electric motor, torque measurement shaft, rolling bearing testing module, flywheel, and load motor.

All tested bearings are of the 6203 type rolling bearings. Experimental data were obtained by installing ball bearings with different damage types into the bearing test module. During the experiment, the sampling frequency for motor current was 64 kHz, for vibration signals, it was 64 kHz, for mechanical parameters (load force, load torque, speed), it was 4 kHz, and for temperature information, it was 1 Hz. Faulty bearings were categorized into artificially induced damage and naturally occurring damage. Artificially induced damage was primarily created using electric sparking (cracks), drilling (spalling), and electric engraving machines (pitting). Naturally occurring damaged bearings were obtained from an accelerated life test rig. Specifically, there were 12 artificially damaged bearings (7 with outer ring damage and 5 with inner ring damage) and 14 bearings damaged through accelerated life testing (5 with outer ring damage, 6 with inner ring damage, and 2 with combined inner and outer ring damage). Additionally, 6 healthy bearings were included in the experiment.

### 5.2. Fault Signal Analysis

The fault characteristic frequencies of the twin data signals and the measured signals under various identical fault modes are compared with the theoretical characteristic frequencies to validate the usability of the twin data signals.

The theoretical fault characteristic frequencies are calculated using the bearing fault characteristic frequency formula. The theoretical fault characteristic frequencies for the 6203 model rolling bearing are shown in Table 3.

**Figure 14:**
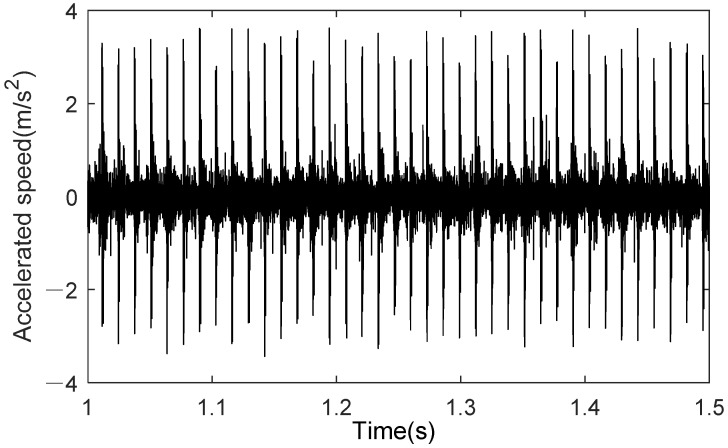
Measured outer ring fault signal.

**Figure 15:**
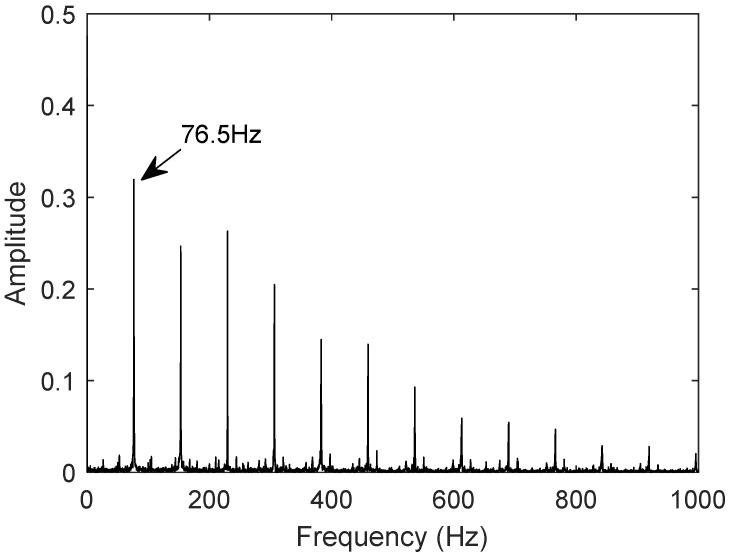
Characteristic frequency of measured outer ring fault signal.

The measured signal uses the bearing dataset N15_M07_F10_KI01_1 from the University of Paderborn, Germany, for the outer race fault. The vibration signal is shown in Figure 14. First, the low-frequency components are obtained using wavelet packet decomposition; then, the envelope is extracted through Hilbert transform, resulting in a denoised signal. Fast Fourier Transform (FFT) is used to generate the frequency-domain plot of the denoised signal. As shown in Figure 15, the fault characteristic frequency of the outer race fault bearing measured signal is 76.5 Hz.

**Figure 16:**
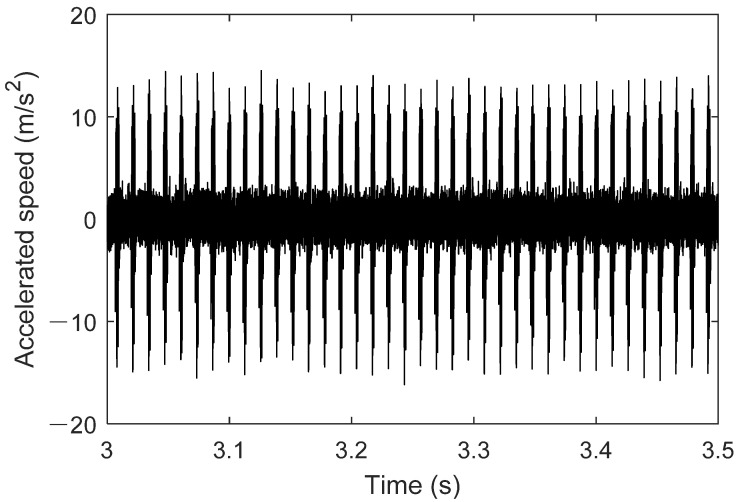
Twin outer ring fault signal.

**Figure 17:**
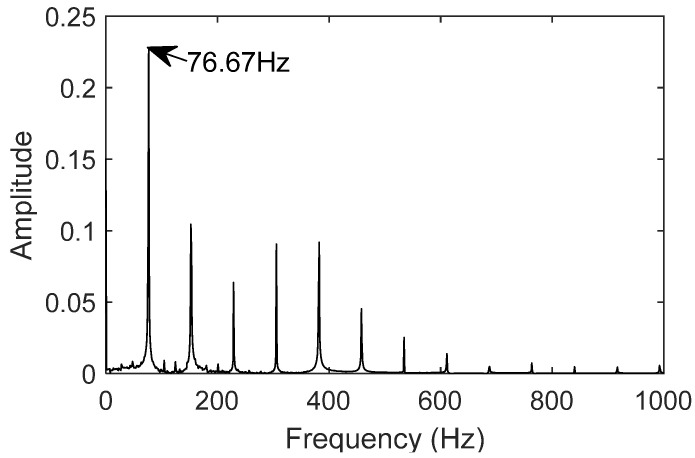
Characteristic frequency of twin outer ring fault signal.

The twin model parameters are adjusted, and the resulting twin data signal for the bearing is shown in Figure 16. The frequency-domain plot of the signal is generated using the Fast Fourier Transform (FFT), as shown in Figure 17, with the fault frequency at 76.67 Hz.

The comparison shows that the error rate between the twin signal and the theoretical value is 0.419%, and between the twin signal and the actual measurement, it is 0.222%. The digital twin model can accurately represent the motion characteristics of the bearing in the outer race fault condition, which aligns with the motion behavior of the bearing under outer race fault conditions.

**Figure 18:**
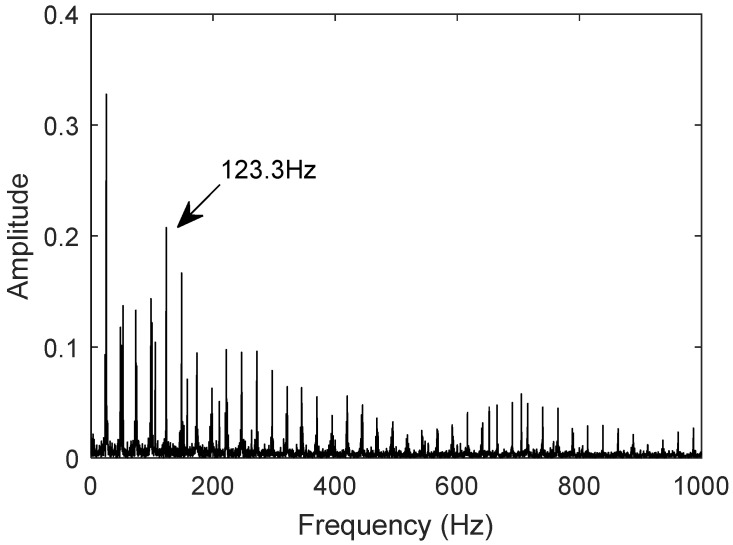
Measured inner ring fault signal characteristic frequency.

**Figure 19:**
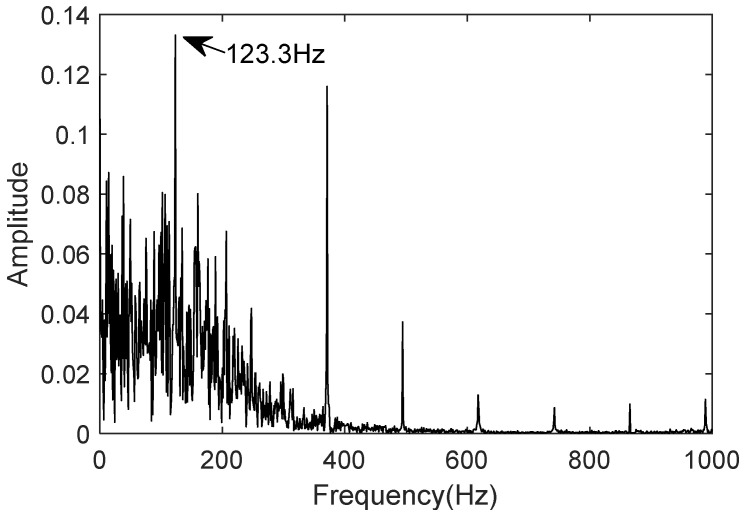
Characteristic frequency of twin inner ring fault signal.

The measured signal uses the bearing dataset N15_M01_F10_KA01_1 from the University of Paderborn, Germany, for the inner race fault. Using the same procedure, the frequency-domain plot of the denoised signal is generated. Figure 18 and Figure 19 show the frequency-domain plots of the measured and twin signals, respectively. The fault characteristic frequency of the inner race fault bearing measured signal is 123.3 Hz, and the fault characteristic frequency of the inner race fault bearing twin signal is also 123.3 Hz.

The comparison shows that the error rate between the twin signal and the theoretical value is 0.276%, and the characteristic frequency of the twin signal matches that of the actual measured value. The digital twin model can accurately represent the motion characteristics of the bearing under inner race fault conditions, which aligns with the motion behavior of the bearing in the inner race fault condition.

### 5.3. Full-Life Signal Analysis

**Figure 20:**
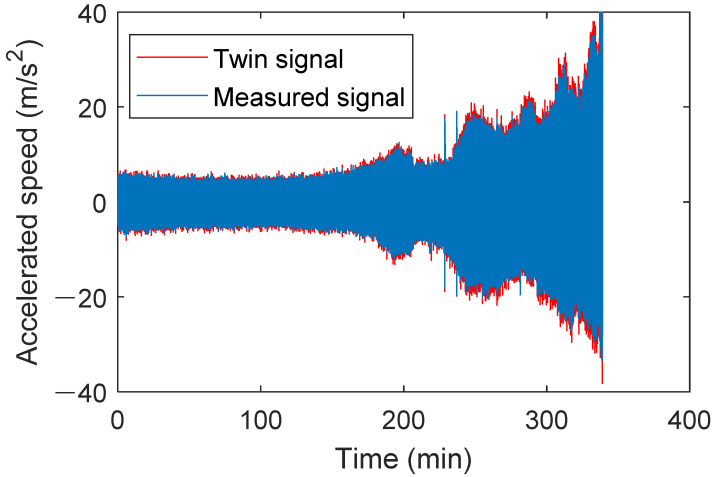
Comparison of the time-domain waveform between the twin signal of bearing 2_5 and the actual signal.

Using data from bearing XJTU-SY 2_5 from the accelerated life test dataset at Xi’an Jiaotong University, the digital twin model built earlier was used to generate the full-life twin signal under this operating condition. First, the parameter degradation curve of bearing defect parameters proposed in this study was used and incorporated into the digital twin model component to update the digital twin model. The rotational speed was set to 2250 rpm, with a sampling frequency of 25.6 kHz, and the degradation type was set to outer race degradation. The full-life degradation signal for the bearing under this operating condition was generated and compared with the measured raw signal, as shown in Figure 20.

**Figure 21:**
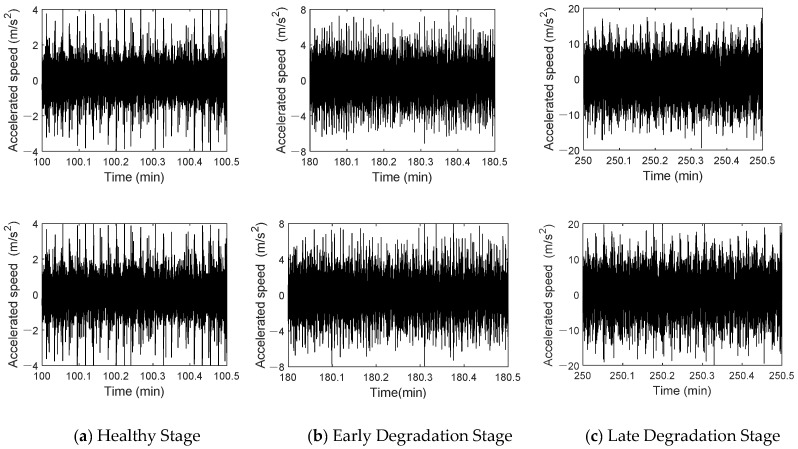
Time-domain waveform comparison between the measured signal and twin signal.

The early degradation stage refers to the initial operating phase of a bearing, where minor wear or performance decline begin to occur, but the bearing can still maintain a relatively stable working condition. The later degradation stage refers to the phase during operation where wear or damage gradually accumulates, leading to a significant decline in performance and approaching failure. Figure 21 shows the time-domain waveforms of the twin signal and the actual signal at different stages. In Figure 21a, the top and bottom graphs represent the time-domain waveforms of the measured and the twin signals during the healthy stage. Figure 21b shows the time-domain waveforms of both signals in the early stage of degradation. Figure 21c illustrates the waveforms of both signals in the late degradation stage. The waveforms of both signals show clear similarity at different stages. No obvious impact characteristics are observed in the normal stage, and the amplitudes are relatively equal. In the early degradation stage, the amplitudes of both signals increase, and the impact characteristics become more evident. In the late degradation stage, dense and obvious impact characteristics appear, and the signal amplitude increases significantly.

It can be concluded through fault signal analysis and full-life signal analysis that the twin signals exhibit very similar time-domain waveforms and fault characteristic frequencies to the actual signals, thereby verifying the accuracy of the constructed digital twin model. This lays a solid foundation for the subsequent use of digital twin signals generated by this method.

## 6. Conclusions

This paper, set against the background of digital twin technology and rolling bearing fault diagnosis, constructs a digital twin integrated model for rolling bearings. Combined with data-driven methods, fault diagnosis for rolling bearings is investigated. The conclusions derived from this study are as follows:

(1) A complete rolling bearing digital twin system is proposed to take rotating mechanical components, specifically rolling bearings, as the research object and integrate digital twin technology. This system mainly consists of the physical entity, digital twin, twin information, data connection, and application layers. The physical entity refers to the bearing testing system of the rotating machinery vibration and fault test bench. At the same time, the digital twin is the rolling bearing integrated model built in Modelica language. A deep-learning-based fault diagnosis model is incorporated as the application layer.
(2) Based on the availability of data and the requirements for constructing the digital twin system, the acceleration vibration signal of the rolling bearing is chosen as the data signal. As one of the most critical components of the digital twin system, the rolling bearing integrated model encompasses bearings in various states, with the bearing drive-end and load-end models included in the digital twin model construction. A hybrid noise component is provided to simulate the bearing’s actual operating state and lifecycle degradation process. Through this model, sufficient and reliable bearing fault data and twin data can be generated.
(3) The German Paderborn University bearing dataset and the XJTU-SY accelerated life test dataset from Xi’an Jiaotong University were used for the experiments. The experimental results show that the digital twin signal verification experiments mainly validate the accuracy and usability of the digital twin signals. By comparing the time-domain waveforms and frequency-domain features of the twin signals and measured signals, it is demonstrated that the twin signals can effectively simulate the vibration characteristics of bearings under fault and degradation conditions. The rational combination of measured and twin data can balance the cost of data acquisition and the model’s prediction accuracy, providing a feasible solution to address data scarcity in practical applications. This demonstrates the feasibility of digital twin technology.

## Figures and Tables

**Table 1:** Monitorable parameters of rolling bearings and their mating parts.

Data Source	Data Source	Data Source
Vibration Sensor	Bearing Vibration Signal	High-frequency Time Series Data
Temperature Sensor	Bearing Temperature Signal	Low-frequency Time Series Data
CNC System	Shaft Speed/Motor Speed	Low-frequency Time Series Data
CNC System	Drive Motor Current	High-frequency Time Series Data

**Table 2:** Basic dimensions of 6205 deep groove ball bearing.

Symbol	Meaning	Value	Symbol	Meaning	Value
D_0_	Bearing outside diameter	39.8 mm	D_i_	Bearing bore diameter	29.3 mm
D	Bearing pitch diameter	34.55 mm	D_b_	Rolling diameter	7.92 mm
B	Bearing width	15 mm	n_b_	Number of rolling elements	8
α	Contact Angle	0°	C	Basic dynamic load rating	12,820 N
C_0_	Basic static load rating	6650 N			

**Table 3:** The theoretical fault characteristic frequency of 6203 deep groove ball bearing (1500 rpm).

Fault Type	Theoretical Fault Characteristic Frequency (Hz)
Outer race damage (BPFO)	76.35
Inner race damage (BPFI)	123.64
Rolling element damage (BSF)	49.92
Cage rotation (FTF)	9.54

## Data Availability

Data are unavailable due to privacy restrictions.
